# T Regulatory Lymphocytes and Endothelial Function in Pediatric Obstructive Sleep Apnea

**DOI:** 10.1371/journal.pone.0069710

**Published:** 2013-07-30

**Authors:** Hui-Leng Tan, David Gozal, Arash Samiei, Rakesh Bhattacharjee, Yang Wang, Helena Molero Ramirez, Hari P. R. Bandla, Richa Kulkarni, Leila Kheirandish-Gozal

**Affiliations:** Section of Pediatric Sleep Medicine, Department of Pediatrics, Pritzker School of Medicine, Biological Sciences Division, The University of Chicago, Chicago, Illinois, United States of America; Imperial College London, United Kingdom

## Abstract

**Background:**

Obstructive sleep apnea (OSA) is a low-grade inflammatory disease affecting the cardiovascular and metabolic systems. Increasing OSA severity reduces T-regulatory lymphocytes (Tregs) in OSA children. Since Tregs modulate endothelial activation, and attenuate insulin resistance, we hypothesized that Tregs are associated with endothelial and metabolic dysfunction in pediatric OSA.

**Methods:**

50 consecutively recruited children (ages 4.8–12 years) underwent overnight polysomnography and fasting homeostatic model (HOMA) of insulin resistance was assessed. Percentage of Tregs using flow cytometry, and endothelial function, expressed as the time to peak occlusive hyperemia (Tmax), were examined. In a subgroup of children (n = 21), in vitro Treg suppression tests were performed.

**Results:**

Circulating Tregs were not significantly associated with either BMI z score or HOMA. However, a significant inverse correlation between percentage of Tregs and Tmax emerged (p<0.0001, r = −0.56). A significant negative correlation between Tregs suppression and the sleep pressure score (SPS), a surrogate measure of sleep fragmentation emerged (p = 0.02, r = −0.51) emerged, but was not present with AHI.

**Conclusions:**

Endothelial function, but not insulin resistance, in OSA children is strongly associated with circulating Tregs and their suppressive function, and appears to correlate with sleep fragmentation. Thus, alterations in T cell lymphocytes may contribute to cardiovascular morbidity in pediatric OSA.

## Introduction

Obstructive sleep apnea (OSA) is characterized by changes in intrathoracic pressure, intermittent hypoxia, hypercapnia and sleep fragmentation. These changes result in sympathetic alterations, increased systemic oxidative stress and the activation of inflammatory processes, all of which likely play important roles in mediating the cardiovascular and metabolic morbidities of OSA [1]–[3].

In adults, longitudinal studies have shown that severe OSA is associated with higher cardiovascular mortality, cardiovascular risk increases in a stepwise fashion with corresponding severity of OSA, and treatment with CPAP reduces both fatal and non-fatal cardiovascular events [4]. Cardiovascular complications have also been described in children with OSA, and include disturbances in blood pressure regulation, ventricular remodeling, and endothelial dysfunction [5]. Endothelial dysfunction is thought to predispose to atherosclerosis and increased cardiovascular risk. Post-occlusive hyperemic response testing has revealed significant impairments in endothelial function among children with OSA when compared to controls, with the majority of these children showing significant improvements in endothelial function after treatment of OSA with adenotonsillectomy [6]. In addition, both obesity and OSA can independently increase the risk of developing endothelial dysfunction, and the concurrent presence of both conditions markedly increases this risk [7], [8]. Markers of inflammation and vascular injury such as adhesion molecules, circulating microparticles and myeloid-related protein 8/14 are all more likely to be increased in children with OSA, and are strongly associated with the presence of endothelial dysfunction [9]–[11]. However, at any given level of OSA severity, not all children with OSA have endothelial dysfunction as assessed by post-occlusive hyperemic responses, suggesting that individual factors underlying the immune response could be responsible for the discrepant vascular manifestations of OSA in children.

Similar to cardiovascular involvement, children with OSA appear to be at increased risk for developing metabolic syndrome [12]. Insulin resistance and alterations in lipid homeostasis have now repeatedly been described in children with OSA, particularly in those who are obese, and inflammatory networks are associated with the degree of metabolic derangement [13], [14].

Most of the research efforts exploring the inflammatory pathways involved in OSA have concentrated on pro- inflammatory immune cells such as monocytes/macrophages and cytotoxic lymphocytes. However, immune homeostasis involves a delicate balance between pro- and anti- inflammatory elements that are tightly regulated, such that reduction in specific anti-inflammatory populations could potentiate inflammation. We have recently reported that increasing OSA severity is associated with a reciprocal decrease in the percentage of T regulatory lymphocytes (Tregs) in the peripheral blood of children with OSA [15]. T regs are a subpopulation of T lymphocytes specialized in immune suppression, that are characterized by their surface markers CD4 and CD25 and expression of the transcription factor Forkhead box protein P3 (FOXP3) [16]. Tregs are critical against inappropriate immune responses, and disruption of their differentiation or function can result in autoimmunity, allergy, inflammation and tumorigenesis [16]. In recent years,

Tregs have been shown to inhibit development and progression of atherosclerosis [17], and affect multiple critical pathways involved in obesity and glucose homeostasis [18]–[20].

Based on aforementioned considerations, we hypothesized that the decrease in Tregs seen in children with OSA contributes to the pathogenesis of endothelial and metabolic dysfunction in the disease. We therefore aimed to determine whether significant associations occur between percentage of circulating Tregs and measures of endothelial and metabolic function, i.e., the postocclusive hyperemic response and the homeostatic model of insulin resistance.

## Methods

Subjects were recruited from the Sleep and ENT clinics of Kosair Children’s Hospital (Louisville) and Comer Children’s Hospital (Chicago), as well as by advertisement. Patients who had genetic or craniofacial syndromes and any chronic diseases such as cardiac disease, diabetes, cerebral palsy and chronic lung disease of prematurity were excluded. The research protocol was approved by the University of Louisville (protocol #474.99) and University of Chicago (protocol 09-115-B) human research ethics committees and written informed consent was obtained from the parents, with assent being obtained from the children.

50 subjects were consecutively recruited, 22 from Louisville, 28 from Chicago. They underwent a standard overnight polysomnography (PSG) as previously described [21], which was scored as per the 2007 American Association of Sleep Medicine guidelines for the scoring of sleep and associated events [22]. The sleep pressure score (SPS), a measure of OSA-induced sleep fragmentation, was calculated using the following equation [23]: SPS = RAI/ARtotI * (1 - SAI/ARtotI) where RAI is the respiratory arousal index, SAI is spontaneous arousal index, ARtotI is the total arousal index.

Height was measured with a stadiometer and recorded to the nearest 0.1 cm. Weight was recorded to the nearest 0.1 kg. Height and weight centiles were calculated using the Centre for Disease Control 2000 growth charts for the United States. BMI z-scores were calculated using The Children’s Hospital of Philadelphia BMI and z-score calculation in children online software (http://stokes.chop.edu/web/zscore).

A fasting blood sample was taken the following morning. Fasting insulin and glucose levels were assayed by the clinical biochemistry laboratory at The University of Chicago Medical Centre. The homeostatic model (HOMA) was calculated using the HOMA calculator software from the University of Oxford as a measure of insulin resistance. The percentage of Tregs was examined using flow cytometry using a Beckman-Coulter Gallios flow cytometer (Beckman Coulter Inc, IL, USA) as described in a previous study [15]. Tregs were defined as cells which were CD4+CD25+ and FOXP3+.

Endothelial function was assessed using a modified hyperemic test as previously described [24]. In brief, this involves occlusion of the radial and ulnar arteries using a blood pressure cuff applied to the forearm. A laser Doppler sensor (Periflux 5000 System integrated with the PF 5050 Pressure Unit, Perimed; Järfälla, Sweden) was applied over the volar aspect of the hand at the distal metacarpal surface of the first finger and the hand was gently immobilized. Tests were performed on awakening in the morning and children were in a fasted state. They lay supine with the head of the couch at 45°. The cuff was connected to a computer controlled manometer and the pressure was raised to 200 mmHg for 60seconds during which blood flow was reduced to undetectable levels. The cuff was then deflated via computer controlled pressure release to allow for consistent deflation times. Hyperemic responses were measured using the laser Doppler device. Commercially available software (Perimed; Järfälla, Sweden) was used to calculate the time to peak regional blood flow post occlusion release (Tmax), a measure of the postocclusion hyperemic response, an index of endothelial function.

**Figure 1:**
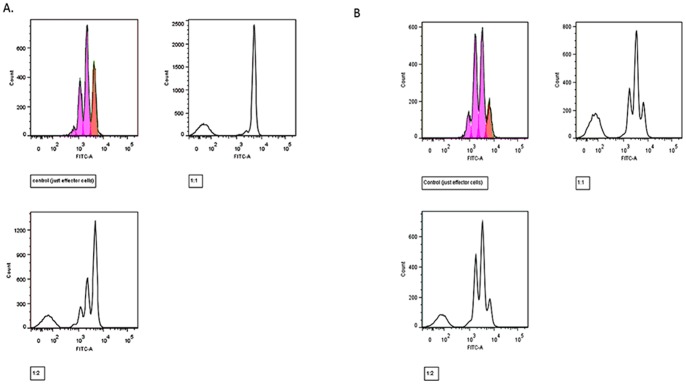
Illustrative examples of Treg immune suppression tests. **a.** Example of T suppression test whereby the Tregs display normal suppressive function. The graphs show proliferation of the effector cells when no Tregs have been added, proliferation of the effector cells when Tregs have been added in a 1∶1 ratio and proliferation of the effector cells when Tregs have been added in a 1∶2 ratio. **b.** Example of T suppression test where the Tregs show poor suppression of effector cell proliferation.

T cell suppression tests [25] were performed in a subgroup of children (n = 21) where sufficient blood was available. CD4+CD25+ and CD4+CD25- cells were first isolated using the RosetteSep® Human CD4+ T Cell Enrichment Cocktail (Stemcell technologies, cat no. #15062) and EasySep® CD25+ Positive Selection Cocktail (Stemcell technologies Human CD25 Positive Selection Kit Catalogue #18231) as per the manufacturers instructions [23]. The purity of the CD4+CD25+ and CD4+CD25- cells was consistently >90%. The CD4+CD25- cells were stained with CFSE CellTraceTM CFSE Cell Proliferation Kit (Molecular probes C34554) for 5 min at room temperature. Staining was quenched with 10 ml PBS with 5%FBS and the cells washed. CD4+CD25+ Tregs were co-cultured with these CD4+CD25- effector cells at varying concentrations [1∶1 (Treg:Teffector), 1∶2, 1∶4 and just CD4+CD25- cells T effector cells] in a 96 well roundbottom plate (30 000 effector cells per well). 2 µl Dynabeads® Human T-Activator CD3/CD28 for Cell Expansion and Activation (Invitrogen cat. no 111-61D) was added to each well. The cells were incubated at 37°C for 4 days in RPMI 1640 medium (Sigma Aldrich), containing 10%FBS, 1 mM sodium pyruvate, 0.3 g/l L-glutamine and Penicillin/streptamycin) and were then analysed using flow cytometry. An example is shown in Figure 1. Treg suppression function was expressed as percentage suppression, calculated using the following equation: 1-(DI 1∶1 ratio/DI only CD4+CD25- effector cells)X100%, DI being the division index, the average number of divisions for all the cells in the original starting population, as calculated using FlowJo software (Treestar Inc, USA). The different ratios were performed to ensure that a dose dependent effect was seen for each sample.

### Data Analysis

Statistical analysis was performed using Graphpad Prism version 5.0 (Graphpad software Inc, CA, USA). When data were normally distributed, they are expressed as mean ± standard deviation and the unpaired t test was used to compare differences between the 2 groups. When data were not normally distributed, they are expressed as median (interquartile range), and the non-parametric Mann Whitney test was used. Categorical data were analyzed using the Chi square test. As T max and SPS data were not normally distributed, non-parametric Spearman’s correlation was used for the calculation of r and p values.

## Results

**Figure 2:**
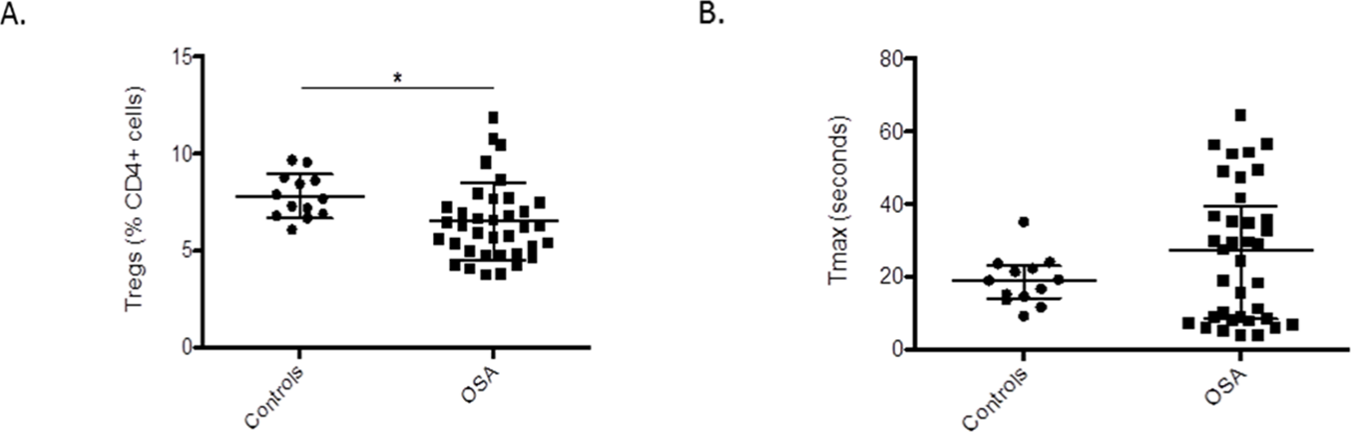
Individual Tregs counts and endothelial function (Tmax) in children with OSA and controls. **a.** Tregs expressed as percentage of CD4+ cells, in children with OSA and controls. **b.** Tmax (time to peak regional blood flow post occlusion release) in children with OSA and controls.

50 subjects were consecutively recruited. They were divided into 2 groups based on their PSG results: controls who had an OAHI<1 (n = 13) and children with OSA (OAHI≥1) (n = 37). Their clinical characteristics and PSG results are summarized in Table 1. The 2 groups were no different with regards to their age, gender, ethnicity and BMI. As expected, the OSA group had a shorter sleep onset latency, spent a shorter period of time in REM sleep, had a higher respiratory arousal index, higher apnea hypopnea index, lower oxygen saturation nadir and spent a higher percentage of time with end tidal CO2>45 mmHg. Children with OSA had a lower percentage of Tregs compared to controls ([mean±SD] [(6.5±2.0) vs. (7.8±1.1) p = 0.03) (Figure 2a). The Tmax of children with OSA was [median(interquartile range)27.5(8.4–39.3)] seconds as compared to 19.0(14.2–23.1)seconds in controls. There was a high degree of variance in the OSA patients (Figure 2b).

**Table 1:** Clinical characteristics and PSG findings in the 50 children studied.

	Controls (AHI<1; n = 13)	OSA (AHI≥1; n = 37)	p value
Age (years)	7.0±1.7	7.7±1.9	NS
Gender (% male) (n)	54% (7)	57% (21)	NS
Ethnicity			NS
%African American (n)	54% (7)	57% (21)	
%White Caucasian (n)	46% (6)	35% (13)	
%Hispanic (n)	0% (0)	5% (2)	
%Others (n)	0% (0)	3% (1)	
BMI z score	1.5±0.7	1.3±1.0	NS
Sleep efficiency (%)	90.1±3.6	88.0±5.9	NS
Sleep onset latency (min)	23.0 (17.8–33.3)	14.0 (7.0–20.0)	p<0.01
REM latency (min)	137.0±45.7	132.4±62.7	NS
Stage 1 (%)	4.5±1.8	6.5±4.6	NS
Stage 2 (%)	42.4±4.4	47.0±8.3	NS
Slow wave sleep (%)	26.3±2.7	25.8±7.4	NS
REM sleep (%)	26.8±5.4	20.8±6.2	p<0.01
Spontaneous arousal index	10.1 (8.9–11.5)	12.3 (8.3–16.3)	NS
Respiratory arousal index	0 (0–0.3)	6.1 (3.1–11.2)	p<0.001
Apnea hypopnea index	0.4±0.4	13.4±12.6	P<0.001
Nadir SpO_2_ (%)	95.7 (92.0–96.8)	87.5 (82.6–90.0)	p<0.001
% time with ETCO_2_>45 mmHg	0 (0–0.6)	3.9 (1.1–9.3)	P<0.001

Normally distributed data are expressed as Mean ± SD; AHI expressed as/hour total sleep time.

Data that are not normally distributed are expressed as Median (interquartile range).

**Figure 3:**
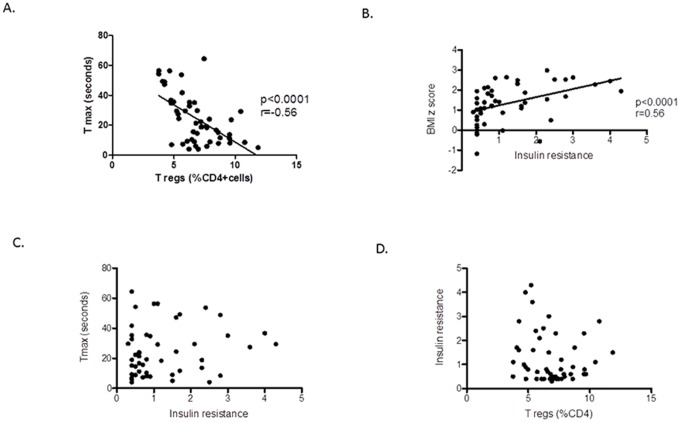
Scatterplots of measures of endothelial function, Tregs, BMI, and HOMA. **a.** Negative correlation between Tmax and circulating Tregs, expressed as percentage of CD4 cells. **b.** Correlation of BMI with HOMA. **c.** There was no correlation of Tmax with HOMA. **d.** There was no correlation between HOMA and circulating Tregs, expressed as percentage of CD4 cells.

Circulating Tregs were not significantly associated with either BMI z score or with HOMA, but were associated with AHI (p<0.01; r: −0.42). However, a strong and highly significant inverse correlation between percentage of Tregs and Tmax emerged (p<0.0001, r = −0.56) (Figure 3a). The lower the percentage of Tregs, the longer it took to reach the peak regional blood flow post occlusion release, implying that children with endothelial dysfunction had lower percentage of Tregs. As expected, HOMA correlated with BMI (p = 0.0003, r = 0.64) (Figure 3b) and obese children had higher insulin resistance. There was no relationship between HOMA and T max (Figure 3c) suggesting that children with insulin resistance do not necessarily have endothelial dysfunction, and therefore, the pathological mechanisms for metabolic impairment and vascular endothelial impairment may be different.

**Figure 4:**
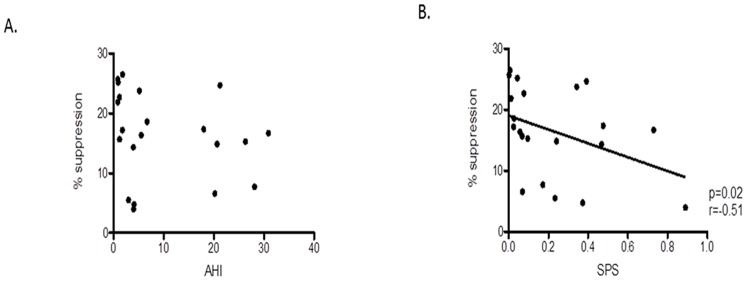
Scatterplots of Tregs suppressive function and 2 polysomnographic measures. **a.** There was no correlation between suppressive function of Tregs with AHI. **b.** Negative correlation between suppressive function of Tregs with the Sleep Pressure Score.

In the subgroup of children who had Treg suppression tests performed, there was no significant correlation between Treg suppressive ability and AHI (Figure 4a). However, a significant negative association between percentage suppression of T lymphocyte proliferation by Tregs and the SPS emerged (p = 0.02, r = −0.51) (Figure 4b).

## Discussion

To the best of our knowledge, this is the first study to indicate that decreased circulating Tregs are associated with endothelial dysfunction in pediatric OSA. When combined with previous data which showed that the degree of decrease in circulating Tregs in children was related to the clinical severity of their OSA, it raises the possibility that the decrease in Tregs may in part mediate the endothelial dysfunction that occurs in a subset of children with OSA. Importantly, this effect appears to be tissue specific, as there is no correlation of Tregs with insulin resistance, confirming the specificity of the former association. The strong association between Tregs suppressive capacity and SPS, but not AHI, suggests that sleep fragmentation rather than intermittent hypoxia may be the primary perturbation affecting Tregs.

The SPS was proposed as a more reliable index of sleep disruption in snoring children than the total arousal index (ARtotI). Tauman et al showed that in a cohort of 559 children, the ARtotI and RAI correlated positively with the AHI [23]. In contrast, the SAI showed an inverse relationship with AHI, possibly because compensatory mechanisms aiming to preserve sleep homeostasis lead to a decline in spontaneous arousals that partially compensate for the reciprocal increase in respiratory arousals. An algorithm incorporating all 3 arousal indices, the SPS, was therefore proposed as a surrogate measure of disrupted sleep homeostasis. Similar findings were reported in adults [26]. When children with high SPS were compared with children with low SPS, they were more likely to have deficits in memory, language abilities, verbal abilities, and some visuospatial functions. The SPS was associated with deficits in neurobehavioral daytime functions, independent of respiratory disturbance and hypoxemia, suggesting a significant role for disturbed sleep homeostasis in pediatric sleep-disordered breathing [27]. The fact that the suppressive capacity of Tregs correlates negatively with SPS, but not with AHI, suggests that sleep fragmentation rather than intermittent hypoxia may be the mechanism by which the observed effects on Tregs are mediated.

Tregs have been linked with endothelial dysfunction and atherosclerosis. Angiotensin II and aldosterone induced blood pressure elevation, vascular oxidative stress, inflammation and endothelial dysfunction can be ameliorated by the adoptive transfer of Tregs [28]–[30]. The increase in systolic blood pressure, impaired acetylcholine vasodilatory responses, increased vascular stiffness, mesenteric artery vascular cell adhesion molecule expression and aortic macrophage and T cell infiltration seen in mice which were infused with Angiotensin II or aldosterone for 2 weeks, were all abrogated when these mice received weekly intravenous infusions of Tregs. IL-10 secreted by Tregs has also been shown to improve microvascular endothelial function in hypertensive mice via inhibition of NADPH oxidase [31].

Endothelial dysfunction is thought to mediate the increased cardiovascular risk seen in patients with OSA, and contribute to the development of atherosclerosis [32]. It is now well established that inflammation plays a key role in the pathogenesis of atherosclerosis [33]. Tregs have been shown to be powerful inhibitors of atherosclerosis in several mouse models [34]. The adoptive transfer of Tregs has been shown to reduce atherosclerotic lesion development and conversely, the depletion of Tregs aggravates lesion development. Intranasal immunization of Apoe^−/−^ mice with an apolipoprotein B-100 fusion protein has been shown to induce antigen-specific Tregs with resultant reduction in aortic atherosclerotic lesion development [35]. It is therefore biologically plausible that the decrease in Tregs seen in pediatric OSA could result in increased inflammation and associated endothelial dysfunction, ultimately leading to atherogenesis.

Tregs have also been linked to obesity and metabolic dysfunction. Leptin, a key hormonal regulator of appetite and metabolism, mainly secreted by adipocytes is thought to be the link between Tregs and obesity [19]. Leptin is a member of the helical cytokine family and has a similar structure to IL-2 [36], a critical T lymphocyte proliferation factor. Leptin is capable of modulating T lymphocyte immune responses, increasing Th1 and suppressing Th2 cytokine production [37]–[39]. Freshly isolated Tregs have been shown to express high amounts of leptin receptor (ObR) and produce substantial amounts of leptin, that result in an autocrine inhibitory loop which constrains their further expansion. Neutralization of leptin in vitro led to the proliferation of Tregs which was IL-2 dependent and FOXP3 expression was also increased [40]. In lean mice, a unique population of Tregs has been shown to reside in adipose tissue and their numbers are reduced in insulin resistant models of obesity [18], where they play a role in the modulation of metabolic parameters. Depletion of these Tregs by injection of diphtheria toxin into mice which expressed diphtheria toxin receptor under the control of FOXP3 transcriptional regulatory components resulted in the induction of several genes encoding inflammatory mediators (including TNFα, IL-6 and RANTES) and the development of insulin resistance. The converse was true in gain of function experiments when the population of Tregs was expanded via injection of a recombinant IL-2, IL-2 specific monoclonal antibody complex [17], [18].^18^ PPAR-γ has recently been shown to be a crucial molecular orchestrator of the accumulation and phenotype of these visceral adipose tissue Tregs [41].

Leptin deficient mice that have a mutation in the leptin gene are overweight, develop severe insulin resistance, and serve as a model for type 2 diabetes and the metabolic syndrome [42]. Induction of Tregs in these mice, by oral administration of anti-CD3 antibody plus β-glucosylceramide, resulted in decreased pancreatic islet cell hyperplasia, decreased inflammation in adipose tissue and lower blood glucose levels [17]. Furthermore, Eller et al showed that the depletion of Tregs in leptin receptor null mice using an anti-CD25 antibody, resulted in increased insulin resistance and impaired insulin sensitivity [20]. Treg depleted mice had more severe diabetic nephropathy, and increased inflammatory cells in their visceral adipose tissue and kidneys compared to leptin receptor deficient mice that were not depleted of Tregs. Conversely, adoptive transfer of Tregs into leptin receptor null mice improved their insulin sensitivity and diabetic nephropathy. Furthermore, these investigators showed that obese patients with insulin resistance display significantly decreased natural Tregs, even though they manifest increased induced Tregs in their visceral adipose tissue when compared to lean control subjects [20]. This could explain the previous conflicting data on Tregs in human visceral adipose tissue of obese patients with and without insulin resistance compared to lean control subjects [43], [44]. Intriguingly, several of the drugs used to treat type 2 diabetes have now been shown to also be immunomodulators. For example, metformin which activates AMP-activated protein kinase (AMPK) and is one of the most common drugs used to treat type 2 diabetes has been shown to inhibit T lymphocyte antigen specific recall responses and the production of Th1 and Th17 cytokines, while upregulating IL-10 secretion in mouse splenocytes [45].

Our findings are consistent with those of other groups, who found no difference in circulating Tregs in children who were obese compared to those who were not [46], [47]. However, some minor differences in gene expression were noted if metabolic syndrome was present [48]. Furthermore, the data we present in this study do not support a relationship between circulating Tregs and insulin resistance in pediatric OSA. A possible explanation is that OSA-induced effects on circulating Tregs are tissue specific, and do not involve mechanisms associated with insulin resistance and metabolic dysfunction, or that longer periods of time are required for Treg effects on pancreatic beta cell function or adipose tissue metabolic dysfunction as elicited by OSA. Alternatively, there is growing realization that the Tregs in adipose tissue have a unique phenotype, different from that of circulating Tregs [48]. It is conceivable that the effects on insulin resistance are mediated by the Tregs in visceral adipose tissue, whereas circulating Tregs play a role in blood vessel endothelial dysfunction. Studies specifically looking at the effect of OSA on Tregs in adipose tissue, leptin levels and insulin resistance would be of clear interest, but are beyond the scope of the current study.

There are some limitations to our study that are worthy of mention so that our findings can be interpreted in the appropriate context: Due to ethical considerations, we were unable to study Tregs in adipose tissues of children. We also performed immunostaining with the standard antibody panel of CD4, CD25 and FOXP3 for the identification of Tregs and future extensive expression profiling with markers such as glucocorticoid induced tumor necrosis factor receptor (GITR), OX40, killer cell lectin-like receptor G2, CD45RA, CD45RO and cytotoxic T lymphocyte antigen 4 (CTLA-4) would be of considerable interest. Furthermore, the study was cross-sectional, so the putative associations identified do not necessarily imply causation. Nonetheless, if we consider Hill’s viewpoints on “what circumstances can we pass from this observed association to a verdict of causation”, the data presented in this study fulfill criteria for strength of association, specificity and biological gradient (dose response), at the same time that the literature discussed above suggests biological plausibility and coherence. There is no doubt that the data from this study are hypothesis generating. Work in animal models is needed to confirm whether the relationship between OSA and decrease in Tregs is indeed a causal one, and to identify the underlying mechanisms of action resulting in vascular end organ morbidity. It has recently been shown, that children with OSA with elevated systemic inflammatory markers, were more likely to have increased DNA methylation of the FOXP3 gene, the unique transcription factor of T regs [49].

It is tempting to speculate that OSA may elicit epigenetic changes responsible for the decrease in Tregs. The resulting decrease in immunosuppressive Tregs may in turn lead to a pro-inflammatory, pro-atherogenic state, a mechanism through which endothelial dysfunction occurs. Furthermore, there is considerable overlap between endothelial dysfunction and neurocognitive deficits in children with OSA [50], and it is not unreasonable to suggest they may well share similar pathogenic mechanisms. Future work exploring the relationship between Tregs and neurocognition would therefore be of considerable interest.

### Conclusions

The presence of endothelial dysfunction in the context of OSA in children is strongly correlated with concomitant changes in circulating Tregs and their function. This suggests that alterations in Treg populations may contribute to cardiovascular morbidity in pediatric OSA. These effects appear to be specific, and do not seem to involve mechanisms underlying insulin resistance. The association between Treg suppressive capacity and SPS, but not AHI, suggests that sleep fragmentation rather than intermittent hypoxia may be the primary perturbation affecting Tregs. Understanding the pathophysiological mechanisms by which OSA results in endothelial and metabolic dysfunction and the variance in phenotype that occurs among different patients is critical, since it would enable risk assessment and optimal interventions in the context of more individualized medical management. Should the decrease/down-regulation in Tregs prove to be a crucial pathogenic pathway for the development of endothelial dysfunction, this may present a novel therapeutic target.
